# SOCIAL MEDIA USE AND MENTAL HEALTH AMONG OLDER ADULTS WITH COMORBIDITIES

**DOI:** 10.1093/geroni/igad104.0268

**Published:** 2023-12-21

## Abstract

Psychological distress is common and problematic for older adults with comorbidities. Social media has great potential to help older adults engage in meaningful social activities and relationships that might improve mental health. However, little information exists about the actual use and the beneficial effects of the use of social media on mental health among this particular population. Using data from the 2019-2020 Health Information National Trends Survey (HINTS), we aimed to describe the prevalence of social media use, and whether social media use is related to better self-care confidence and thus related to better mental health among U.S. older adults with comorbidities. Data were analyzed using weighted logistic models and mediation analyses that considered the complex survey design with jackknife replications. Variables were assessed by self-reports. The prevalence of social media use increased rapidly from 41% in 2017 to 54% in 2020 among U.S. older adults with comorbidities. Those who were of younger age, women, higher education, or higher income were more likely to use social media, whereas those who were Hispanic or Black were less likely to use social media. Social media use was related to better self-care confidence (b=0.17, 95% CI=0.07-0.26, P< 0.001) and is further related to better mental health (b=1.07, 95% CI=0.86-1.28, P< 0.001). Our results suggest that social media is a beneficial supplement to traditional sources of social support for mental health. However, more attention is needed to those who are not only affected by commodities but also socially disadvantaged (e.g., Hispanic or Black with lower education).

